# Recent Findings and Developments in Spine Biomechanics

**DOI:** 10.3390/bioengineering13040482

**Published:** 2026-04-21

**Authors:** Christian Liebsch

**Affiliations:** Institute of Orthopaedic Research and Biomechanics, Ulm University Medical Centre, 89081 Ulm, Germany; christian.liebsch@uni-ulm.de

## 1. Introduction

As the central musculoskeletal element of the human body, the spine simultaneously enables trunk movement, upright posture, and load transfer from the upper to the lower body. Consequently, the spine must withstand a variety of forces and moments and exhibit unique material properties and kinematics. However, the fundamental importance of the spine for human biomechanics is also accompanied by multiple musculoskeletal spinal pathologies, with spine-related pain being one of the main causes of disability worldwide. A more detailed knowledge of spinal biomechanics is therefore essential regarding the prevention and treatment of musculoskeletal spinal diseases. To contribute to this, the Special Issue “Spine Biomechanics” aimed to collate new findings and developments in biomechanical research of the spine, comprising in vivo (clinical) trials, in vitro studies, and numerical modelling studies on the spine, the design and validation of novel research methodologies for spinal biomechanics, and investigations of novel technologies and devices for the orthopaedic and traumatological treatment of the spine, as well as studies on the effects of influencing factors on spinal biomechanics, such as ageing, degeneration, and trauma.

## 2. In Vivo (Clinical) Trials

In recent years, evolving technologies regarding in vivo measurements of spinal kinematics have resulted in an increasing number of studies investigating intervertebral motion in both healthy subjects and specific patient groups. These data are not only valuable for the understanding of normal spine function; they can also serve as input and validation data for numerical simulations and experimental models and may support clinical diagnostics as well as the assessment of surgical outcomes. In their scoping review, Breen et al. identified six categories of research questions being addressed by in vivo studies analysing lumbar spinal motion, comprising ‘normal biomechanical mechanisms’, ‘direct kinematic measurement’, ‘dynamic radiography’, ‘pathological and injury mechanisms’, ‘spinal stabilisation’, and ‘clinical markers’, thus proposing a novel taxonomy for in vivo measurements of spinal kinematics [1]. Concerning clinical markers, the prospective cross-sectional study of Hoehl et al. provided a unique dataset comprising both structural and functional changes in participants suffering from chronic or intermittent low back pain as well as participants without any back pain [2]. In this study, Hoehl et al. found that chronic and intermittent low back pain were both associated with lower rates of high physical activity as well as with more prevalent morphological and functional impairments compared to painlessness, respectively, indicating the requirement for a more accurate differentiation between specific types of low back pain and their potential causes and therapeutic consequences. Regarding acute low back pain, the retrospective clinical trial of Löchel et al. was furthermore able to show that patients with lumbar disc herniation at the L4–L5 level exhibit significantly different spinopelvic anatomy in terms of the clinical parameters of pelvic incidence, pelvic tilt, and relative lumbar lordosis, as well as the absolute difference between pelvic incidence and lumbar lordosis compared to patients with lumbar disc herniation at the L5–S1 level [3]. The findings of this study indicate that spinopelvic anatomy represents a relevant factor for the segmental level where lumbar disc herniations occur, potentially affecting the biomechanical load distribution in the lower lumbar spine, which should be further investigated by future in silico studies. In a longitudinal pilot study, Rossi et al. furthermore found indications for relationships between asymmetry in the neurodynamic function of the brachial plexus, determined by the Upper Limb Neural Tension Test, and upper thoracic spine kinematics, quantified by a novel motion analysis technique [4]. In particular, the authors detected altered motion patterns in lateral bending and proposed the use of manual therapy to potentially address both the neurodynamic and biomechanical impairments—a suggestion which should be substantiated in any follow-up studies. Furthermore, in their systematic literature review, Yoseph et al. concluded that about half of all pregnant women are predisposed to low back pain due to increased lumbar lordosis, ligamentous laxity, altered gait mechanics, and muscular deconditioning [5]. Moreover, they determined associations between these pregnancy-related biomechanical changes and sacroiliac joint laxity, anterior pelvic tilt, and multiparity, as well as potential long-term risks of degenerative disc disease and spondylolisthesis from previous studies, highlighting the clinical relevance of spinal biomechanics in maternal spinal health.

## 3. In Vitro Studies

Experimental studies investigating spinal biomechanics are essential to directly address clinical questions related to the primary and long-term stability of spinal implants and to quantitatively assess the potential effects of pathologies and surgical approaches on spinal flexibility and kinematics. In vitro studies generally have the advantage of being able to evaluate novel treatment options under standardised and thus reproducible loading conditions, which are essential for the objective assessment of their clinical applicability. One common issue in this context is the testing of implants and implant combinations in cases of reduced bone quality. The study of Jacob et al. investigated whether a novel screw augmentation technique using two additional subcortical screws would be able to prevent cage subsidence when performing Transforaminal Lumbar Interbody Fusion [6]. Compared to the control group, who underwent surgery using the conventional cage placement technique, higher failure loads as well as a higher number of load cycles until reaching cage subsidence were found, indicating the advantages of the novel technique regarding long-term stability. However, different failure modes also implied potential complications related to the novel approach, requiring further investigations. Another clinical challenge in the context of reduced bone quality is the treatment of osteoporotic vertebral body fractures. In cases of severe wedge-shaped morphologies of the fractured vertebral body, causing kyphotic spinal deformity, the so-called kyphoplasty procedure is usually performed to straighten the affected vertebra. The findings of Riesenbeck et al. revealed that two-compartment kyphoplasty was not significantly superior in height reconstruction and load-bearing capacity compared to one-compartment kyphoplasty and showed that higher cement volume correlated with the occurrence of adjacent vertebral fractures [7]. Moreover, Riesenbeck et al. determined a significant reduction in post-traumatic segmental instability when applying kyphoplasty; however, this did not lead to complete restoration of native spinal stability [8]. Another clinically relevant problem in spine surgery, often associated with reduced bone quality, is pedicle screw loosening. In addition to these studies investigating the potential effects of bone quality and pedicle morphology on the anchorage capacity of pedicle screws, screw malpositioning is also widely discussed in the literature as a potential influencing factor for pedicle screw loosening. In their study, however, Schleifenbaum et al. were able to prove that minor deviations in screw placement had no major effect on the fixation strength of pedicle screws [9]. As the main clinically relevant consequence of pedicle screw malpositioning is the potential injury caused to adjacent structures, particularly the spinal cord, Kurz et al. investigated whether screw malpositioning can be prevented during the insertion process by directly measuring the screw insertion resistance [10]. However, using standardised testing conditions, Kurz et al. did not find any evidence that torque-based mechanical resistance is a reliable and consistent real-time indicator of pedicle screw malposition. Another important aspect in clinical spinal biomechanics is the kinematic behaviour of motion-preserving implants, especially in the cervical spine. To better understand the complex motion characteristics of disc prostheses under in vivo-like movements, Ansaripour et al. developed a novel testing method to simulate combined rotational–translational motions [11]. Validating their test setup, Ansaripour et al. could show that simultaneous rotation–translation motion provoked subluxation of ball-and-socket prostheses at lower motion extents compared to isolated motion types.

## 4. In Silico Investigations

Compared to in vitro studies, which can also provide input and validation data for numerical models of the spine, finite element analyses offer insights into load distributions, stress concentrations, and local strains in both spinal structures and implants, offering risk estimations regarding potential tissue and implant failure following surgical treatment and allowing direct comparisons between surgical techniques under the exact same boundary conditions. In the study of Dhar et al., this principle was used to compare the traditional bilateral pedicle screw–rod system with a novel reverse transdiscal screw system, which can be applied for the minimally invasive surgical treatment of lumbar degenerative disc disease [12]. Findings of this study revealed that the use of the reverse transdiscal screws resulted in lower segmental range of motion, lower screw and cage stresses, and higher anterior and posterior shear load resistance compared to the use of the pedicle screw–rod system. In a similar study, Lodde et al. investigated the effectiveness of different fixation techniques for the treatment of unilateral non-displaced fragility fracture of the pelvis [13]. Lodde et al. could show that both bilateral iliosacral and transsacral screws produced less dislocation and local stress concentrations compared to unilateral iliosacral screw fixation, with bilateral iliosacral screws providing higher rotational stability compared to transsacral screws. Apart from the evaluation of implant performance, finite element models of the spine can be used to investigate alterations in the load distribution in cases of spinal deformity and degeneration. Wang et al. used finite element analysis to determine stresses in the discs adjacent to spinal fixation following scoliosis treatment [14]. Deriving parametrised finite element models from biplanar pre- and post-operative X-ray images of an adolescent idiopathic scoliosis patient, Wang et al. determined a decrease in maximum endplate and disc stress in the cranial adjacent segment as well as an increase in stress in the caudal adjacent segment following spinal fixation, indicating a high risk for adjacent segment disease below scoliosis instrumentation. Using another parametric finite element model, Ardatov et al. investigated the impact of progressing disc degeneration on lumbar spinal biomechanics by gradually adjusting disc height, nucleus pulposus volume, and tissue stiffness to simulate altered dehydration and fibrosis [15]. Applying displacement-controlled compressive loading, Ardatov et al. could show that anulus fibrosus stresses largely increased while nucleus pulposus stresses as well as intradiscal pressure values decreased, indicating significant load redistribution during the process of disc degeneration, including a shift in primary compressive load absorption from the nucleus pulposus to the anulus fibrosus.

## 5. Design and Validation of Novel Methods

New and further developments of methods to investigate spinal biomechanics are essential to answer unacknowledged or complex research questions related to the spine. Advancements and innovations in the field of engineering and programming enable novel, more detailed, and highly specific designs of experimental and numerical models. Remus et al. developed a muscle-driven numerical model of the human torso within the open-source modelling framework ArtiSynth, combining forward dynamic simulation with finite element modelling, enabling them to study interactions between active and passive spinal structures and spinal implants as well as orthoses under physiologically realistic loading conditions [16]. Using this model, investigations of loads inside spinal instrumentation, intervertebral discs, and surrounding soft tissue can be performed under physiological loading conditions, allowing studies on clinical phenomena such as adjacent segment degeneration as well as facilitating predictions of treatment outcomes. Apart from basic and translational research, the design and validation of novel diagnostic methods are of major importance for their potential clinical use. Kniepert et al. introduced Dynamic Surface Topography as a novel technique to analyse spine kinematics in patients with back pain under dynamic conditions in an easy-to-use, radiation-free setup [17]. Validating this novel method in a prospective observational study, Kniepert et al. observed more movement in patients with more pain and concluded that Dynamic Surface Topography can provide valuable therapeutic information for an individual patient. In another study, Konradi et al. showed that Surface Topography proves extensive inter-individual variability as well as high intra-individual consistency of spinal motion during gait [18]. To enable replication and validation of their findings, Konradi et al. made all tools and visualisations freely available in repositories. Finally, van Santbrink et al. presented a novel, artificial intelligence-assisted technique for the image recognition of cervical vertebrae in dynamic X-ray recordings in order to investigate qualitative motion patterns in the cervical spine [19]. Using a U-Net architecture, van Santbrink et al. could prove the feasibility of implementing deep learning models to detect qualitative cervical motion patterns and thus found promising approaches for the use of this technique in clinical research.

## 6. Conclusions

Research on spinal biomechanics has been constantly growing due to progressive advancements in methodology and technology, and will further diversify due to the necessity to address upcoming topics related to the ageing society and novel diagnostic and therapeutic strategies in orthopaedics and traumatology. While in vivo studies have been shown to allow for increasingly precise measurements of three-dimensional spinal motion and the detection of specific motion patterns influenced by spinal pathology, injury, and pain, in vitro studies enable quantifications of spinal flexibility, kinematics, and intra-articular pressure under standardised loading conditions, which are essential for implant testing and biomechanical evaluations of novel surgical treatment strategies, as well as the generation of input and validation data for numerical models of the spine. The number of finite element analyses for the investigation of spinal biomechanics is constantly increasing, as relevant information regarding stress and strain distributions inside anatomical structures and implants that cannot be directly quantified from in vivo and in vitro studies is discovered. Future in silico studies will presumably use more complex musculoskeletal models, comprising inverse and forward dynamics in addition to finite element modelling, in order to investigate spinal biomechanics under more physiologically realistic loading conditions.

## References

[1] Breen A., Breen A., Branney J., du Rose A., Nematimoez M. (2026). Advances in the Measurement and Interpretation of Intervertebral Motion in the Lumbar Spine: A Scoping Review. Bioengineering.

[2] Hoehl B.U., Taheri N., Schonnagel L., Becker L.A., Modl L., Reitmaier S., Pumberger M., Schmidt H. (2025). Comprehensive Analysis of Chronic Low Back Pain: Morphological and Functional Impairments, Physical Activity Patterns, and Epidemiology in a German Population-Based Cross-Sectional Study. Bioengineering.

[3] Löchel J., Hanisch M., Burger J., Labbus K., Zahn R. (2025). Association of Spinopelvic Anatomy with the Level of Lumbar Disc Herniation. Bioengineering.

[4] Rossi M., Signorini M., Baram A., De Robertis M., Capo G., Riva M., Fornari M., Pessina F., Brembilla C. (2025). Upper Limb Neural Tension Test and Spinal Biomechanics: Insights from a Longitudinal Pilot Study. Bioengineering.

[5] Yoseph E.T., Taiwo R., Kiapour A., Touponse G., Massaad E., Theologitis M., Wu J.Y., Williamson T., Zygourakis C.C. (2025). Pregnancy-Related Spinal Biomechanics: A Review of Low Back Pain and Degenerative Spine Disease. Bioengineering.

[6] Jacob A., Feist A., Zderic I., Gueorguiev B., Caspar J., Wirtz C.R., Richards G., Loibl M., Haschtmann D., Fekete T.F. (2025). Augmenting Screw Technique to Prevent TLIF Cage Subsidence: A Biomechanical In Vitro Study. Bioengineering.

[7] Riesenbeck O., Czarnowski N., Raschke M.J., Oeckenpohler S., Hartensuer R. (2024). Biomechanical Comparisons between One- and Two-Compartment Devices for Reconstructing Vertebrae by Kyphoplasty. Bioengineering.

[8] Riesenbeck O., Czarnowski N., Raschke M.J., Oeckenpohler S., Hartensuer R. (2024). Primary Stability of Kyphoplasty in Incomplete Vertebral Body Burst Fractures in Osteoporosis: A Biomechanical Investigation. Bioengineering.

[9] Schleifenbaum S., Metzner F., Schultze J., Kurz S., Heyde C.E., Pieroh P. (2025). Does Malpositioning of Pedicle Screws Affect Biomechanical Stability in a Novel Quasistatic Test Setup?. Bioengineering.

[10] Kurz S., Fischer B., Schultze J., Metzner F., Wendler T., Heyde C.E., Schleifenbaum S. (2025). Does Resistance Indicate Malposition? A Standardized Comparison of Pedicle Screw Placement. Bioengineering.

[11] Ansaripour H., Ferguson S.J., Flohr M. (2024). Evaluation of Load on Cervical Disc Prosthesis by Imposing Complex Motion: Multiplanar Motion and Combined Rotational-Translational Motion. Bioengineering.

[12] Dhar U.K., Aghayev K., Sultan H., Rajendran S., Tsai C.T., Vrionis F.D. (2025). Finite Element Analysis of Biomechanical Assessment: Traditional Bilateral Pedicle Screw System vs. Novel Reverse Transdiscal Screw System for Lumbar Degenerative Disc Disease. Bioengineering.

[13] Lodde M.F., Klimek M., Herbst E., Peez C., Riesenbeck O., Raschke M.J., Rosslenbroich S. (2025). Bilateral Iliosacral and Transsacral Screws Are Biomechanically Favorable and Reduce the Risk for Fracture Progression in Fragility Fractures of the Pelvis-A Finite Element Analysis. Bioengineering.

[14] Wang T.H., Chou P.H., Chen C.S. (2025). Using Two X-Ray Images to Create a Parameterized Scoliotic Spine Model and Analyze Disk Stress Adjacent to Spinal Fixation-A Finite Element Analysis. Bioengineering.

[15] Ardatov O., Fernandes S.R., Kilikevicius A., Alekna V. (2026). Parametric Finite Element Evaluation of Load Redistribution Under Progressive Lumbar Disc Degeneration. Bioengineering.

[16] Remus R., Lipphaus A., Ritter M., Neumann M., Bender B. (2025). A Muscle-Driven Spine Model for Predictive Simulations in the Design of Spinal Implants and Lumbar Orthoses. Bioengineering.

[17] Kniepert J., Ronsch H., Betz U., Konradi J., Huthwelker J., Wolf C., Westphal R., Drees P. (2025). Dynamic Surface Topography for Thoracic and Lumbar Pain Patients-Applicability and First Results. Bioengineering.

[18] Konradi J., Betz U., Huthwelker J., Wolf C., Schmidtmann I., Westphal R., Cerpa M., Lenke L.G., Drees P. (2025). Using Surface Topography to Visualize Spinal Motion During Gait-Examples of Possible Applications and All Tools for Open Science. Bioengineering.

[19] van Santbrink E., Schuermans V., Cerfonteijn E., Breeuwer M., Smeets A., van Santbrink H., Boselie T. (2025). AI-Assisted Image Recognition of Cervical Spine Vertebrae in Dynamic X-Ray Recordings. Bioengineering.

